# Effects of transcutaneous electrical nerve stimulation on pain after impacted third molar surgery

**DOI:** 10.4317/medoral.22871

**Published:** 2019-04-24

**Authors:** Ahmet-Taylan Çebi

**Affiliations:** 1 Karabük University Faculty of Dentistry, Department of Oral and Maxillofacial Surgery

## Abstract

**Background:**

The aim of this study was to assess the therapeutic effects of Transcutaneous Electrical Nerve Stimulation (TENS) on pain after the impacted third molar surgery.

**Material and Methods:**

The study was carried out on 30 patients attending the outpatient department of the oral and maxillofacial surgery. Both sides’ impacted mandibular third molars were taken into consideration, and a total of 60 impacted third molars were undergone surgical extraction. After the first surgery, patients were given analgesic, antibiotics, and mouthwash as a routine treatment procedure. Following the second surgery, TENS was applied over the painful area of the cheek for patients in addition to routine treatment procedure. Pain was evaluated with Visual Analog Scale (VAS) at the postoperative 6, 8, 24 hours and 2,3,4 and 5 days. The Student t test was applied to compare pain levels between groups. *P*<0.05 and *p*<0.001 were considered significant in all statistical analyses.

**Results:**

The study was carried out in 30 patients 15 (50%) female and 15 (50%) male, aged between 20 and 31 years. The mean age of the patients was 24.60 ± 4.76. There was a statistically significant difference in the evaluation of VAS levels in postoperative 6, 8, 24 hours and 2, 3, 4, and 5 days between the routine treatment group and the TENS application group.

**Conclusions:**

TENS activates a complex neuronal network to result in a reduction in pain. In conclusion, TENS application was highly effective in pain modulation following the third molar surgery. Therefore, TENS, which is one of the non-pharmacological pain control methods after such surgeries, can be used safely in reducing postoperative pain.

** Key words:**Impacted teeth, third molar, pain, transcutaneous electrical nerve stimulation.

## Introduction

Surgical extraction of the impacted teeth is one of the most frequently performed procedures in oral surgery (1-3). Surgical interventions are a major cause of pain. After a short time from surgical extraction, postoperative pain may occur (3,4). Pain is one of the most common problems experienced in the postoperative period. After the impacted teeth operations, the pain starts after the loss of effects of anesthesia and increases to the maximum level in the first 6-12 hours after the operation (4,6). It has been reported that pain is affected negatively the quality of life of patients (5,6). Therefore, postoperative pain control is very important for the comfort of patients.

Many pharmacological and non-pharmacological methods are used in order to reduce the severity and the amount of complications such as pain, swelling, and trismus which are developed after the impacted third molar surgery (7).

Transcutaneous electrical nerve stimulation (TENS) is a non-pharmacological pain control method based on the method of applying different frequency electrical currents via the surface electrodes placed on the skin and activates a complex neuronal network to reduce pain by activating descending inhibitory systems in the central nervous system to reduce hyperalgesia. This method, which is cheap, safe and easy to apply, can be used in addition to pharmacological agents in the treatment of postoperative pain (8,9).

Previous reports show that TENS reduces pain through both peripheral and central mechanisms. Centrally, sites in the spinal cord and brainstem that utilize opioid, serotonin, and muscarinic receptors are activated by TENS. At the site of TENS application, opioid and α-2 noradrenergic receptors are peripherally involved in TENS- induced analgesia (10).

The aim of this study was to evaluate the efficacy of TENS on postoperative pain after mandibular third molar surgery.

## Material and Methods

The study was conducted in accordance with the guidelines on the Helsinki Declaration on Human Rights. The study was started after obtaining permission from the Ethics Committee of Karabük University (Date: 29.03.2017, Decision Number:3/10). This randomized clinical study was performed with 30 patients. Patients between the ages of 20 and 31 years who presented for surgical extraction of mandibular bilateral third molars to the Oral and Maxillofacial Surgery Clinics were included in the study. The number of patients to be included in the study was determined as the result of power analysis. The patient group included in the study consisted of systemically healthy individuals and thirty patients with bilateral impacted mandibular third molar in the vertical position and bone retention. Exclusion criteria from the study were; infection of the surgical region, renal or hepatic disease, pregnancy or lactation, heart disease and patients with pacemaker, known hypersensitivities, sensitivities, or reactions to any of the medicines and smoking or alcohol addiction. All the patients were given detailed information about the study and consent form was taken from all of the patients.

In this study, 60 impacted third molar surgeries were performed under local anesthesia at 30 patients.

-Surgical procedure;

Surgeries of the bilateral impacted third molars were completed under local anesthesia (2% articaine hydrochloride with 1:100,000 adrenaline) after sufficient height and impression of the buccal mucoperiosteal flap. İmpacted third molars extraction were performed under irrigation of physiologic saline (0.9%). The mucoperiosteal flap was repositioned and sutured. The mean duration of operation was 20-25 minutes.

Patients were given a day 2x1 and every day the same hours and during 5 days, flurbiprofen 100 mg (Majezik, Sanovel, Turkey), amoxicillin (Augmentin 625 mg 14 tablets Glaxo Smith Kline Pharmaceuticals Industry and Trade Co.) and chlorhexidine gluconate %2 (Chlorhex Gargle 200ml, Drogsan Pharmaceutical Industry) after the first surgery procedure. After the second surgery, the same medical procedure was applied and TENS (50HZ frequency, 100-microsecond short pulse duration) was applied for 15 minutes daily for 5 days postoperatively at the same time every day. Wollex WX-2120 (Ruian Tianlun Health Equipment Co., Zhejiang, China) was used as the TENS device. The TENS electrodes were placed on the skin of the angulus and corpus of the mandible over the surgical area by the researcher. All surgeries and TENS application were performed by an oral and maxillofacial surgeon (the researcher).

-Measurement of pain;

In the evaluation of postoperative pain intensity, 10 cm Visual Pain Scale (VAS) was used which was filled at 6, 8, 24 hours and 2,3,4 and 5 days after the operation. Scores 0-3 reported mild pain, 4-7 reported moderate pain and reported 8-10 severe pain.

The statistical analyses were performed using the statistical package ‘Minitab 17’. Relevant data collected in the study were analyzed using descriptive statistics of means and standard deviations. The Student t test was applied to compare pain levels between groups. The chi-square test was compare gender. *P* <0.05 and *p* <0.001 were considered significant in all statistical analyses.

## Results

The study was carried out in 30 patients 15 (50%) female and 15 (50%) male, aged between 20 and 31 years. The mean age of the patients was 24.60 ± 4.76 ( Table 1). There was no statistically significant difference between the mean age and sex distributions of the groups ( Table 1).

**Table 1:**
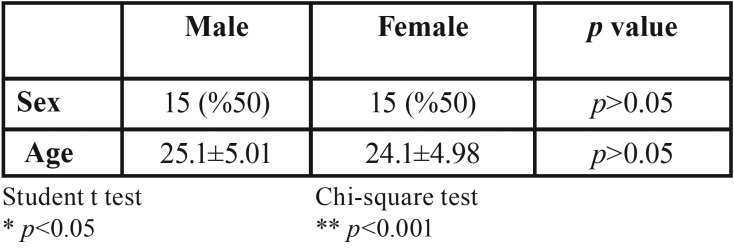
Comparison of sex and ages.

There was a statistically significant difference between VAS levels at the 8th and 24th hours after the first surgery which included routine procedure between male and female patients. Pain levels of female patients were found to be higher at 8th and 24th hours *p*<0.05, *p*<0.001) ( Table 2).

**Table 2:**
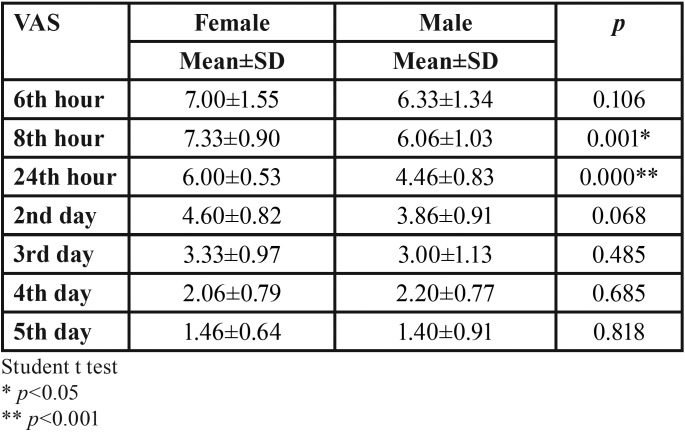
Comparison of VAS levels between female and male after routine post-op surgery treatment procedure.

There was no statistically significant difference between the VAS levels of female and male patients after the second operation which was applied TENS (*p*>0.05) ( Table 3).

**Table 3:**
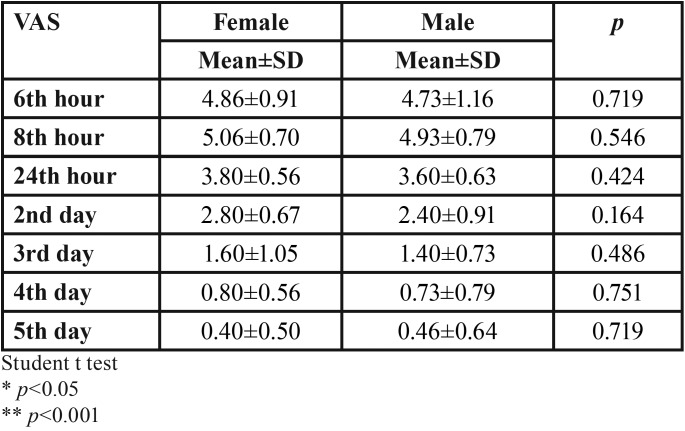
Comparison of VAS levels between female and male after post-op TENS.

There was a statistically significant difference in the evaluation of VAS levels in postoperative 6, 8, 24 hours and 2, 3, 4, and 5 days between the routine surgery group and the TENS group (*p* <0.001) ( Table 4). TENS application was found to be significantly effective on postoperative pain.

**Table 4:**
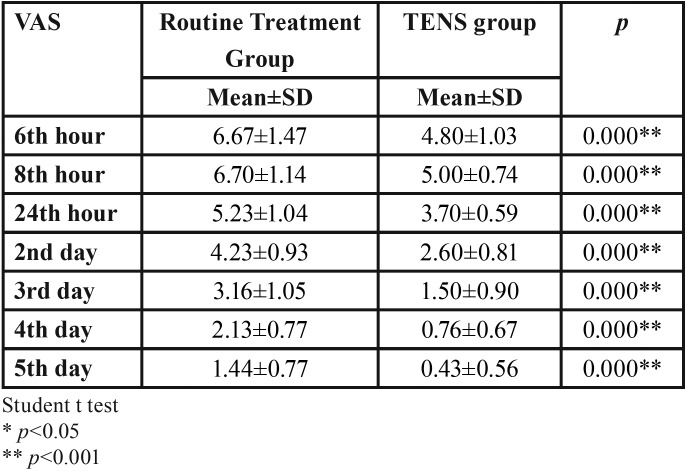
Comparison of VAS levels between routine treatment group and TENS group after third molar surgery.

## Discussion

Postoperative complications such as pain, edema, trismus, bleeding, infection, nerve damage, and fracture may occur after the surgical extraction of the impacted third molar teeth in oral and maxillofacial surgery (5,6). These complications may have a negative impact on the life comfort of the patients postoperatively (6,11). In our study, we evaluated the effectiveness of TENS application to the level of pain which is the most common complication that affects the patient comfort at the highest level after the third molar surgery.

In our study, postoperative pain was first evaluated at 6 hours postoperatively. The reason for this is that the half-life of flurbiprofen used in the study is 6 hours and the most severe postoperative pain occurs after the first 6-8 hours (12,13).

Neal *et al.* (14) used a 100 mm VAS to assess the pain after tooth extraction in their study and Boonstra *et al.* (15) used a 10 cm VAS in the evaluation of pain. In our study, the 10 cm VAS was used in the pain assessment after the third molar dental surgery.

Fisher *et al.* (16) and Parry *et al.* (17) reported that female felt more postoperative pain than male. In our study, it was found that after the impacted third molar surgery, the pain levels of female patients were higher than in male patients.

TENS is a non-pharmacological pain control method that is cheap, safe, non-invasive and without serious side effects. It is easily tolerated by patients and can be easily administered by the patients themselves (18). For these reasons, we evaluated the effectiveness of TENS in pain after the impacted third molar surgery in our clinical study.

Strassburg *et al.* pulled teeth without local anesthesia in their study. Furthermore, TENS was applied to patients during tooth extraction. They evaluated the efficacy of TENS application on acute pain occurring during tooth extraction and indicated that it was an effective method for pain (19). Johnson et al. reported that TENS application, which is a non-pharmacological pain control method, is effective on acute pain (20). In another study, TENS has been reported to be effective on pain during orthodontic tooth movements (21). Walsh *et al.* have not been able to obtain a definite result on the effectiveness of TENS application on acute pain (22). As a result of our study, TENS application was found to be effective on postoperative pain.

Olaogun *et al.* evaluated the effectiveness of TENS application on pain, edema, and trismus after the third molar surgery and reported that TENS application was effective on pain as a result of their studies (23). Arabion *et al.*, divided the patients into two groups after surgery and they gave only one dose of analgesic to one group after surgery, while the other group performed TENS with a single dose analgesic. They performed pain assessment with VAS at the postoperative 8th hour and found that the pain level was lower in the TENS treated group (24). In our study, the efficacy of TENS application on postoperative pain after the impacted third molar surgeries were evaluated and it was concluded that TENS application was highly effective on postoperative pain.

## Conclusions

The results of this study supported previous studies on the analgesic effect of TENS. Furthermore, the study revealed that TENS were also effective in pain control after the impacted third molar surgery. Use of TENS may decrease pain level following impacted third molar surgery and also, the need for additional doses of nonsteroidal anti-inflammatory drugs.
